# Pre-operative education and prehabilitation provision for patients undergoing hip and knee replacement: a national survey of current NHS practice

**DOI:** 10.1186/s12891-025-08637-5

**Published:** 2025-04-29

**Authors:** Ifrah Omar, Vikki Wylde, Jodie Fogg, Michael Whitehouse, Wendy Bertram

**Affiliations:** 1 Bristol Medical School, University of Bristol, Bristol, UK; 2 National Institute for Health and Care Research Bristol Biomedical Research Centre, University Hospitals Bristol and Weston NHS Foundation Trust and the University of Bristol, Bristol, UK; 3 National Institute for Health and Care Research (NIHR) Applied Research Collaboration West, Bristol, UK

## Abstract

**Background:**

Over 215,000 total hip replacements (THRs) and total knee replacements (TKRs) take place annually in the UK. Joint replacement has the longest waiting times of elective surgical treatments, with some patients waiting up to two years for surgery in the NHS. Pre-operative education and support interventions could improve both pre-operative health and optimise post-operative outcomes. However, current pre-operative NHS service provision is heterogenous and poorly described. This study aimed to describe the current services and care provided to patients on NHS waiting lists for THR and TKR.

**Methods:**

A link to a national online survey about pre-operative education and prehabilitation was sent to relevant healthcare professionals involved in the pre-operative care of patients waiting for THR or TKR surgery at a sample of high-, mid- and low-volume NHS hospitals in the UK. Participants were asked questions about what pre-operative education and pre-habilitation services were offered at their hospital, and any barriers or facilitators to delivering care. Frequency statistics were used to describe categorical data and free-text data were coded into categories.

**Results:**

Responses were received from 29 UK hospitals across seven regions. Pre-operative education was provided to patients at 28 hospitals, primarily at single session talks supplemented with booklets. Prehabilitation was provided to patients waiting for TKR at 17 hospitals and to patients waiting for THR at 14 hospitals. It comprised strengthening exercises and advice with written information. Three hospital respondents did not know if prehabilitation was provided before TKR and four hospitals before THR. Barriers to service provision include funding, staffing, facilities, and lack of awareness/evidence on how best to deliver services.

**Conclusions:**

Prehabilitation services are not provided for all patients waiting for arthroplasty. Future work is needed to design and evaluate prehabilitation resources to optimise pre-operative health and improve patient outcomes after TKR and THR.

**Supplementary Information:**

The online version contains supplementary material available at 10.1186/s12891-025-08637-5.

## Background

Over 200,000 total hip replacements (THR) and total knee replacements (TKR) are performed every year in the UK [1, 2]. The aim of joint replacement is to reduce chronic pain and improve functional ability. Since 2023, around 3.25 million NHS patients have been waiting more than 18 months for elective treatment [3]. Elective orthopaedic surgery has the longest waiting times with some patients waiting eighteen months or longer for joint replacement [4–6]. Due to long waiting lists, millions of patients are living with severe pain, reduced mobility, poor mental health and a loss of independence [7]. Patient health-related quality of life deteriorates when waiting from six to twelve months for joint replacement [8]. Approximately 20–40% of patients on the waiting list for joint replacement are living in a health state worse than death [6, 9].

Pre-operative care can play a key role in managing pain, function and general health while patients wait for elective joint replacement surgery. First, the strongest risk factor for chronic pain after joint replacement, which affects approximately 10–20% of patients, is pre-operative pain severity [10–14]. Pre-operative interventions that reduce pain severity therefore have the potential to optimise longer-term post-operative pain outcomes. Second, pre-operative education can provide patients with confidence in preparing for surgery, reassurance about hospital discharge, set realistic expectations about recovery and shorten the length of hospital stay [15–18]. Third, pre-operative exercise, or prehabilitation, has been shown to improve function after THR and TKR surgery and reduce length of stay [18]. There are published recommendations for the content and delivery of education and prehabilitation before TKR [19]. Getting It Right First Time (GIRFT) is an NHS England programme which provides best practice evidence-based guidance for both hip and knee replacement pathways, and specifies provision of prehabilitation, patient information and education before surgery [20, 21]. However, little is known about service provision in the NHS. A top 10 research priority from the James Lind Alliance priority setting partnership for hip and knee replacement for osteoarthritis is to understand what health services can influence post-operative outcomes [22]. Mapping current service provision can identify gaps in service provision and provide important contextual information for future research.

The aim of this national survey was to describe the education and prehabilitation currently provided to patients waiting for hip and knee replacement at NHS hospitals.

## Methods

This national survey was conducted as a Clinical Effectiveness Project with approval from North Bristol NHS Trust (CE93612).

Using data from the National Joint Registry (https://www.njrcentre.org.uk/healthcare-providers/statsonline/), 259 NHS centers performing THRs and TKRs in England, Wales and Northern Ireland were identified. For the purposes of sampling, hospitals were classified into high-volume, medium-volume and low-volume. High-volume hospitals were defined as hospitals performing ≥ 252 TKRs between April 2022 to March 2023; mid-volume hospitals were those that performed 50–251 TKRs in the same period, and low-volume hospitals were defined as those performing under ≤ 50 TKRs in that time period.

The first 86 hospitals by volume in each group were sampled for the survey and attempts were made to identify contact details of staff or teams that would be familiar with pre-operative service provision for patients waiting for THR and TKR. Contacts were identified using contact details provided on the hospital website or by telephoning the hospital. Where contacts at the hospital/Trust were not identified, the researchers contacted local research teams identified from the NHS R&D Contacts directory to forward details on. Contact details for 79 high-volume, 74 mid-volume, and 67 low-volume hospitals were identified. Contacts were sent an email with a brief introduction and a link to the online survey. A reminder email was sent to non-responders one week after initial contact. Those who did not respond two weeks after the reminder email were considered non-responders.

No sample size calculation was performed for this study. In terms of sampling, the aim was to gain response from a range of different volume hospitals and geographic areas to understand variation in service provision. A target sample size of 30 hospitals was chosen pragmatically as an achievable response rate based on previous similar national surveys of service provision [23–25].

The survey questions were developed by the research team, informed by previous national surveys of rehabilitation services for primary TKR [26], rehabilitation services for revision TKR [24], care pathways for chronic pain after TKR [27], and consensus work on pre-operative education and prehabilitation for TKR [19]. Questions were primarily multiple choice with the option to provide further detail in free-text boxes. The full survey is provided in Appendix 1. The survey was piloted with clinical members of the research team (a physiotherapist and orthopaedic surgeon) prior to being administered. The survey consisted of 40 questions across five sections, covering pre-operative education (separate sections for TKR and THR), prehabilitation (separate sections for TKR and THR) and suggestions for potential improvements to service provision and barriers to implementation of these improvements. Respondents were also asked to provide their hospital name and job title. Completed surveys were anonymous at the level of the respondent. The survey was administered using Online Surveys (www.onlinesurveys.ac.uk).

### Patient and public involvement (PPI)

The survey was designed in collaboration with the University of Bristol’s Musculoskeletal Research Unit’s Patient Experience Partnership in Research (PEP-R) group. PEP-R is a group of nine patients with musculoskeletal conditions, most of whom have experience of hip or knee replacement surgery. The group felt that pre-operative education and prehabilitation was an important topic and said patients should be given information to help themselves. This includes the need to maintain muscle strength to ensure better post-operative recovery and that exercises should be given to help with this. They observed that pool-based exercises and hydrotherapy are often suggested as gentle exercises that can be carried out with minimum pain; however, these exercises are not accessible to everyone. The members felt strongly that patients on the waiting list for joint replacement should be contacted and asked about their physical health, medication and well-being and that it is important to let people know they are still on the waiting list and where they are on the list. They also suggested that information should be provided to patients about other methods of addressing pain to make their day-to-day lives more manageable, such as transcutaneous electrical nerve stimulation (TENS) machines, heat, braces, and arch supports. They suggested a video as a helpful way of sharing this information.

To incorporate PEP-Rs suggestions into the survey specific items about the provision of pain management, use of equipment/aids, education videos, and hydrotherapy were included in the questionnaire.

### Analysis

The survey responses were exported to Excel for analysis. Duplicate responses from different staff at the same hospital were reviewed by two members of the research team for completeness and agreement. There were no contradictory responses among the duplicates. The most complete response from each hospital was selected for inclusion in the analysis. In one case, where one respondent completed the education section only and another completed the prehabilitation section, the responses were merged into a single record. Frequency statistics were used to describe categorical data and free-text data were coded into categories. Descriptive summaries of each service were developed through this process. All individual responses to the free-text questions about suggested improvements in service provision were used in the analysis. Free-text responses were coded by two authors using content analysis to develop a descriptive summary, using methods described in a previous survey study of service provision [24].

## Results

### Participants

The survey was open from July-September 2023. A total of 41 responses were received (response rate of 19%) from hospitals in England and Wales. Of these, 20 were duplicate responses from staff at the same hospital (5 hospitals with 2 responders and 2 hospitals with 3 responders and 1 hospital with 4 responders). After merging of these duplicate responses, the final dataset consisted of responses from 29 hospitals (13%, *n* = 29/220). Characteristics of respondents and hospitals are provided in Table 1.

**Table 1:** Characteristics of survey respondents

Responder characteristics	Number
**Hospital volume**	
High	16
Medium	7
Low	6
**Hospital geographical region**	
South East England	9
South West England	4
North East England	5
North West England	2
East Midlands England	4
West Midlands England	3
Wales	2
**Profession**	
Physiotherapist	16
Orthopaedic Team Lead/Manager	4
Nurse	3
Occupational Therapist	3
Orthopaedic Surgeon	1
Health Care Assistant	1
Anaesthetist	1

### Pre-operative education

Pre-operative education was provided to patients waiting for TKR and THR by 28 of 29 hospitals. One hospital did not provide patients with education before undergoing TKR. One hospital respondent did not know whether patients were provided with education before undergoing THR. Fifteen hospitals provided the same education to patients listed for THR and TKR surgery. The education delivered to patients listed for THR and TKR differed at 13 hospitals. Details of education provision and content for patients waiting for TKR and THR are provided in Table 2. Education was primarily provided in a single face-to-face session with talks/presentations led by nurses, physiotherapists and occupational therapists, with provision of written information/booklets. Some hospitals offered virtual, video, website or other electronic formats. Timing of delivery was primarily between consent and/or the pre-operative assessment appointment and surgery. Standardised education was offered at nine hospitals, with 18 offering additional support/signposting where needed. One hospital provided education for frail patients only. Content was varied, most commonly including expectations of pain and recovery, what to expect during the hospital stay and following hospital discharge, rehabilitation after surgery, making home preparations and common issues after surgery, returning to daily activities, use of equipment and aids, and pain management.

**Table 2:** Pre-operative education provision for patients listed for TKR and THR

Pre-operative education survey topic	TKR, Number of hospitals *n* = 28	THR, Number of hospitals *n* = 28
Education setting*		
Face-to-face	23	22
Virtual	7	7
Booklet or other written format	23	23
Video or DVD	8	7
Website or other electronic format	13	11
Timing of Education*		
At the point of listing	6	4
Between consent and/or pre-operative assessment appointment and surgery	19	16
Between listing and consent and/or pre-operative assessment appointment	9	7
Don’t know	5	3
**Standardised or Tailored Education**		
Standardised only	9	10
Standardised, but additional support/signposting is provided where needed	18	15
Tailored	1	2
**Delivery over single or several sessions**		
Single	19	18
1–2 sessions	4	5
More than 2 sessions	3	3
Don’t know	2	1
Health professionals delivering sessions*		
Nurse	18	17
Occupational Therapist	14	18
Physiotherapist	24	24
Psychologist	0	0
Orthopaedic Surgeon	11	11
Pharmacist	0	0
Anaesthetist	4	4
Don’t know	2	1
Other: Band 4, Therapy Assistant Practitioner	2	3
Other: Nutritionist	0	1
Delivery format*		
Talks/presentations	22	20
Videos/DVDs	11	10
Website/app	12	12
Written information	23	22
Don’t know	1	1
Other: Individualised as required	1	2
Content*		
Making home preparations	22	23
What to expect during the hospital stay	24	25
What to expect following discharge	23	26
Risks of surgery and how to minimise them	19	19
Common issues that may occur after surgery which do not need to cause alarm	24	21
Organising help if complications occur	10	9
Arranging social support	14	15
Recovery expectations	25	25
Returning to daily activities	21	25
Returning to driving and other types of travel	23	24
Returning to work	18	20
Returning to sport/leisure activities	15	18
Pain expectations	22	23
Pain management	21	23
Pain science education	3	5
Rehabilitation after surgery	25	25
Purpose of pre-habilitation	17	18
Physical activity	15	18
Weight management	10	11
Weight loss	6	6
Swelling	21	20
Smoking/alcohol cessation	13	12
Nutrition	13	12
Goal setting	13	12
Use of equipment/aids	21	21
Anatomy	13	14
Don’t know	3	1
Other: Hip precautions, leg length measurement	0	2

*Respondents could provide more than one response

### Prehabilitation

For patients waiting for TKR, 17 hospitals provided prehabilitation, nine hospitals did not provide prehabilitation and three respondents did not know if prehabilitation was provided. Of the 17 hospitals that provided prehabilitation, eight provided prehabilitation to all patients waiting for TKR and nine provided prehabilitation to patients who met certain criteria, most commonly referral by their surgeon or GP or based on frailty, patient history or having a city postcode.

For patients waiting for THR, 14 hospitals provided prehabilitation, 11 hospitals did not provide prehabilitation and four respondents did not know if their hospital provided prehabilitation. Of the 14 hospitals that provided prehabilitation for patients waiting for THR, 10 provided the same prehabilitation as for patients waiting for TKR, two respondents were not aware if the prehabilitation differed for patients waiting for TKR and THR and two hospitals provided different prehabilitation for THR. Differences in prehabilitation at these two hospitals were that written information was provided to TKR patients but not THR patients, THR patients were provided with an unsupervised home exercise programme (one hospital), and different treatment modalities and exercises were used (both hospitals provided ice/heat to THR patients but not TKR patients, one hospital provided occupational therapy to THR but not TKR patients, and one hospital provided pain management to TKR but not THR patients).

Details of the prehabilitation provided are provided in Table 3. A summary and comparison of prehabilitation provision for patients waiting for TKR and THR is provided in Table 4.

**Table 3:** Prehabilitation provision for TKR and THR

Survey topic	TKR, Number of hospitals (*n* = 17)	THR, Number of hospitals (*n* = 14)
**Timing of prehabilitation provision**		
Between listing and consent/pre-operative assessment	4	3
Between consent/pre-operative appointment and surgery	7	3
Between listing and surgery	3	3
When referred/requested	2	1
Don’t know	1	4
**Location**		
Hospital outpatients	9	6
Hospital outpatients and home	3	3
Home	2	1
Community	1	1
Don’t know	2	3
Format*		
Written information	13	8
Individual session	9	6
Group-based class	8	6
Unsupervised home exercise programme	8	7
Telephone/ video call	4	4
Home visit	1	1
Don’t know	1	2
**Number of treatment sessions**		
< 2	4	2
2 to 4	1	2
5 to 6	2	1
> 6	2	2
Tailored to individual patient	1	1
Don’t know	7	6
Treatment modalities*		
Exercise	14	10
Advice	13	10
Weight programme referral	9	7
Pain management (including CBT)	5	3
Occupational therapy	5	5
Ice/Heat	4	5
Manual therapy (including soft tissue techniques)	4	3
Nutrition	3	2
Hydrotherapy	3	3
Electrotherapy	0	0
Acupuncture	0	0
CBT for formally diagnosed depression or anxiety	0	0
Don’t know	1	2
Types of exercise* (n = 14)		
Strengthening	12	9
Walking practice with walking aids	9	7
Flexibility	8	5
Practicing post-operative exercises	7	5
Balance	7	6
Functional movement (including gait re-education)	7	4
Training on steps	6	4
Functional technique	5	4
Core control	4	4
Cardiovascular	2	3
**Provision of aids/specific equipment**		
No	4	3
Yes -provided by hospital	7	5
Yes– provided by outside provider as part of a trial	1	0
Don’t know	5	6

*Respondents could provide more than one response

**Table 4:** Summary of prehabilitation provision for TKR and THR patients

Survey topic	Number of hospitals
**Provision of prehabilitation to TKR and THR patients**	
Prehabilitation for both TKR and THR patients	12
No prehabilitation for TKR or THR patients	8
Prehabilitation for TKR patients only	3
Prehabilitation for THR patients only	1
Prehabilitation unknown for THR and/or TKR patients	5

Timing of prehabilitation provision was variable, with some hospitals delivering prehabilitation between listing and consent/pre-operative assessment and others between consent/pre-operative assessment and surgery; two hospitals provided prehabilitation when referrals/requests were received. Prehabilitation was most commonly provided in the hospital, although some hospitals provided prehabilitation that could be undertaken in a home setting. Written information was the most common format for prehabilitation, followed by individual sessions, group-based sessions and unsupervised home exercises. The number of treatment sessions varied from a single session to more than six. A wide range of treatment modalities were used, most frequently exercise and advice, and referral to weight management programmes was common. Types of exercise were also varied, and commonly included strengthening, walking practice with aids, flexibility, practicing post-operative exercises, balance and functional movement. Eight hospitals provided specific equipment or aids for patients waiting for TKR and five for THR.

### Improvements to service provision

Suggestions for improvements to service provision were considered from all respondents, including duplicates from the same hospital. Of the 41 responders to the survey, 27 identified a total of 37 areas for improvement in the provision of services for pre-operative patients, which were coded into ten categories (Table 5). The most commonly suggested areas for improvement were provision/reinstatement of joint schools offering a comprehensive group based multi-session programme of prehabilitation and education, prehabilitation provision for all patients undergoing joint replacement and more comprehensive prehabilitation provision. Barriers to these services being provided were funding (11 respondents), staffing (9 respondents), facilities (6 respondents), lack of awareness/evidence on how best to deliver services (4 respondents), patients experiencing barriers to accessing virtual resources (1 respondent) and services not being restarted after the COVID-19 pandemic (1 respondent).

**Table 5:** Areas for pre-operative service improvement

Improvements to service provision	Number of respondents
Provision/reinstatement of joint schools	10
Prehabilitation provision for all patients undergoing joint replacement	8
More comprehensive prehabilitation provision e.g. medical, waiting well programme, social services, OT, nutrition, blood analysis, hydrotherapy, bariatric services	7
Alternative delivery methods e.g. apps, virtual, home visits	4
Earlier/extended access to prehabilitation services	2
More consistency in care e.g. between surgical teams, gym referrals	2
Fewer patients going to independent treatment centres	1
Evaluation of effectiveness of prehabilitation	1
Multidisciplinary input into joint schools	1
Individualised care e.g. education	1

## Discussion

Pre-operative education is offered before THR and TKR surgery in face-to-face and booklet format by most hospitals taking part in this study. Prehabilitation provision is less prevalent, with around half of hospitals offering prehabilitation. Suggested improvements to services included the provision of joint schools and more comprehensive prehabilitation offered to all patients. Barriers to delivery include funding, staffing, facilities and lack of awareness/evidence on how best to deliver services.

In addition to the evidence supporting improved outcomes with pre-operative education and prehabilitation, it is an important topic to patients. Pre-operative education, support, and advice features in the top 10 of the James Lind Alliance Priority Setting Partnership for Hip and Knee Replacement for osteoarthritis [28]. Underserved communities place importance on access to physiotherapy services, exercises for joint pain, and self-management of joint pain and symptoms while waiting for joint replacement [29].

### Limitations

The survey received responses from 29 hospitals covering seven UK regions, with a good spread across high, middle and low volume centres. The response rate to the survey is a limitation, with only 29 hospitals represented out of the 220 hospitals contacted. Although the findings do not represent a national picture of service provision, they provide an overview of service provision, including barriers and facilitators, as well as potential improvements. Scotland and Northern Ireland are not represented in the responses and the low response rate limits the external validity of the conclusions that can be drawn. Data collection for this survey took place from July to September, which may have contributed to a lower response rate due to summer holidays.

Some staff responding to the survey were not able to answer all the questions. Data on staff seniority or number of years in profession was not collected in order to keep the survey as short as possible and decrease burden on participants. Although this information might provide insight into the responder’s background, this survey was primarily focused on service provision. We are aware that in several hospitals, there is a separate hip and knee service with no overlap in clinicians, which may account for staff not being aware of work taking place outside of their subspecialty.

### Pre-operative education

Pre-operative education is more often delivered in a single session and not tailored to individual patient needs. Psychological input is not provided, despite factors such as anxiety, depression, and catastrophizing being known predictors for poor post operative outcomes [30, 31]. Lack of tailoring further increases health inequalities for patients with multiple health conditions. Individualised care for these patients may reduce post-operative emergency department attendances [32]. Pre-operative education appears to focus on basic education and expectations rather than optimising health conditions which affect outcomes, and may contribute to surgical cancellations [33, 34].

### Pre-habilitation

The prehabilitation provision of 17/29 hospitals for TKR and 14/29 for THR reflects findings from a recent freedom of information request study reporting provision of prehabilitation for orthopaedic surgery at 55% of hospitals [35]. Prehabilitation is not currently comprehensive enough and should be tailored to individual patient abilities and needs. Outside of the UK, the literature focusses primarily on effectiveness, rather than provision, of arthroplasty prehabilitation. However, one survey of older patients in the Netherlands found that about 40% received a prehabilitation programme before arthroplasty [36]. Targeted and multimodal prehabilitation for frail patients waiting for joint replacement surgery may have beneficial effects on physical function, general health and post-operative outcomes, but further evidence is needed [37–39].

There is evidence that patients receiving prehabilitation prior to joint replacement have a decreased length of stay and improved function compared with patients who do not [40, 41]. Despite the known benefits and inclusion in the NHS England GIRFT recommended pathways, this survey indicates that many NHS hospitals do not provide prehabilitation to patients waiting for THR and TKR. Longer waiting lists have resulted in deteriorating patient health and function while waiting for surgery [8, 9]. Many joint school programmes providing education and prehabilitation were cancelled during the COVID-19 pandemic and have not been reinstated. Digital or virtual joint schools have been introduced and show some improvements in outcomes [42, 43]. However, this may not be appropriate for all patients in the demographic undergoing joint replacement, raising the issue of digital exclusion for the 38.8% of adults aged 75 and over who have never used the internet [44].

### Implications

Our aim was to determine what the current provision was and whether this aligned with the evidence base on optimising outcomes for patients undergoing hip and knee replacement. Where the two do not align, we feel it is important that this is identified so that service delivery can be appropriately planned, resourced and delivered to optimise patient experience and outcomes. NHS staff and services are under increased pressures, with a backlog of patients on the waiting list for hip and knee replacement that will take years to resolve [45]. This raises the need for research to explore innovative healthcare delivery methods, such as artificial intelligence and digital platforms, which could support patients while reducing the burden on NHS staff.

## Conclusion

In conclusion, this survey of a small sample of NHS hospitals has highlighted a lack of provision of tailored education and prehabilitation before TKR and THR, and highlighted areas for service improvement, including barriers and facilitators. Future work is needed to design and evaluate tailored education and prehabilitation interventions for patients waiting for THR and TKR to optimise pre-operative health and post-operative outcomes, with consideration given to addressing the potential barriers to implementation within an NHS setting.

## Electronic supplementary material

Below is the link to the electronic supplementary material.

Appendix 1

## Data Availability

The data sets generated during the present study will be available in the University of Bristol Research Data Repository (https://data.bris.ac.uk/). Data will be available within 6 months following publication. Access to the data will be restricted to ensure that data are only made available to bona fide researchers for ethically approved research projects, on the understanding that confidentiality will be maintained and after a data access agreement has been signed by an institutional signatory.
