# Supplementary material for: Pre-operative education and prehabilitation provision for patients undergoing hip and knee replacement: a national survey of current NHS practice
Source: BMC Musculoskelet Disord. 2025 Apr 29;26:421. doi: 10.1186/s12891-025-08637-5 (PMC12039115; doi:10.1186/s12891-025-08637-5)
Supplement: Supplementary file 1 — Appendix 1 [file 12891_2025_8637_MOESM1_ESM.docx]

**Support for patients on the waiting list for hip and knee replacement: A survey of current practice**

**Thank you for helping us with our service evaluation project, which aims to identify what services are currently provided for patients waiting for hip and knee replacement in NHS hospitals around the UK.**

There are up to 39 questions across 5 sections in the survey, and we expect it will take you about 15-20 minutes to complete. Any answers you give today will be kept anonymous and will not be directly linked to you or your hospital in any reporting of the survey findings.

**About You**

1. Which hospital/trust do you work for?
2. What is your job title? *[Required]*
3. We are conducting this survey to help identify areas of priority for future research and develop interventions to optimise the health of patients waiting for hip and knee replacement. Would you be interested in being contacted about taking part? *[Required]*

- Yes
  - Please leave your contact details (name and email/phone) below: *[Required]*
- No

**Section 1: Pre-operative education for patients waiting for knee replacement**

1. At your hospital, are patients provided with education before undergoing knee replacement? *[Required]*

- Yes
- No *[skip to Section 2]*
- Don’t know *[skip to Section 2]*

**S1.1: Pre-operative education for patients waiting for knee replacement**

1. In what setting is the education provided? (Please tick all that apply)

- Face to face
  - Please specify ‘Face to face’: *[Required]*
    - Pre-operative assessment clinic
    - Knee class/school
    - Home visit
    - Outpatient
- Virtual (video or telephone)
  - Please specify ‘Virtual (video or telephone): *[Required]*
    - Video call
    - Telephone call
- Booklet or other written format
- Video or DVD
- Website or other electronic format
- Don’t know
- Other
  - Please specify ‘Other’: *[Required]*

1. When is the education delivered?

- At the point of listing
- Between listing and consent and/or pre-operative assessment appointment
- Between consent and/or pre-operative assessment appointment and surgery
- Don’t know
- Other
  - Please specify ‘Other’: *[Required]*

1. Is the education standardised for everyone or tailored to individuals? over a single or several sessions?

- Standardised only
- Standardised, but additional support/signposting is provided where needed (please provide details)
- Tailored
  - Please provide details: *[Required]*

1. Is the education delivered over a single or several sessions?

- Single
- 1-2 sessions
- More than 2 sessions
- Don’t know

1. Which health professionals are involved in providing the pre-operative education? (Please tick all that apply)

- Nurse
- Occupational therapist
- Physiotherapist
- Psychologist
- Orthopaedic surgeon
- Pharmacist
- Anaesthetist
- Don’t know
- Other
  - Please specify ‘Other’: *[Required]*

1. How is the education provided? (Please tick all that apply)

- Talks/presentations
- Videos/DVD
- Website/app
- Written information
- Don’t know
- Other
  - Please specify ‘Other’: *[Required]*

1. What is the content of the pre-operative education? (Please tick all that apply)

- Making home preparations
- What to expect during the hospital stay
- What to expect following discharge
- Risks of surgery and how to minimise them
- Common issues that may occur after surgery which do not need to cause alarm
- Organising help if complications occur
- Arranging social support
- Recovery expectations
- Returning to daily activities
- Returning to driving and other types of travel
- Returning to work
- Returning to sport/leisure activities
- Pain expectations
- Pain management
- Pain science education
- Rehabilitation after surgery
- Purpose of pre-habilitation
- Physical activity
- Weight management
- Weight loss
- Swelling
- Smoking/alcohol cessation
- Nutrition
- Goal setting
- Use of equipment/aids
- Anatomy
- Don’t know
- Other
  - Please specify ‘Other’: *[Required]*

**Section 2: Pre-operative education for patients waiting for hip replacement**

1. At your hospital, are patients provided with education before undergoing hip replacement? *[Required]*
   - Yes
   - No *[skip to Section 3]*
   - Don’t know *[skip to Section 3]*

**S2.1: Pre-operative education for patients waiting for hip replacement**

1. Is the pre-operative education provided to patients while on the waiting list for hip replacement **different** to that provided to patients waiting for knee replacement? *[Required]*
   - Yes
   - No *[skip to Section 3]*
   - Don’t Know *[skip to Section 3]*

**S2.2: Pre-operative education for patients waiting for hip replacement**

1. In what setting is the education provided? (Please tick all that apply)

- Face to face
  - Please specify ‘Face to face’: *[Required]*
    - Pre-operative assessment clinic
    - Knee class/school
    - Home visit
    - Outpatient
- Virtual (video or telephone)
  - Please specify ‘Virtual (video or telephone): *[Required]*
    - Video call
    - Telephone call
- Booklet or other written format
- Video or DVD
- Website or other electronic format
- Don’t know
- Other
  - Please specify ‘Other’: *[Required]*

1. When is the education delivered?

- At the point of listing
- Between listing and consent and/or pre-operative assessment appointment
- Between consent and/or pre-operative assessment appointment and surgery
- Don’t know
- Other
  - Please specify ‘Other’: *[Required]*

1. Is the education standardised for everyone or tailored to individuals?

- Standardised only
- Standardised, but additional support/signposting is provided where needed (please provide details)
- Tailored
- Please provide details: *[Required]*

1. Is the education delivered over a single or several sessions?

- Single
- 1-2 sessions
- More than 2 sessions
- Don’t know

1. Which health professionals are involved in providing the pre-operative education? (Please tick all that apply)

- Nurse
- Occupational therapist
- Physiotherapist
- Psychologist
- Orthopaedic surgeon
- Pharmacist
- Anaesthetist
- Don’t know
- Other
  - Please specify ‘Other’: *[Required]*

1. How is the education provided? (Please tick all that apply)

- Talks/presentations
- Videos/DVD
- Website/app
- Written information
- Don’t know
- Other
  - Please specify ‘Other’: *[Required]*

1. What is the content of the pre-operative education? (Please tick all that apply)

- Making home preparations
- What to expect during the hospital stay
- What to expect following discharge
- Risks of surgery and how to minimise them
- Common issues that may occur after surgery which do not need to cause alarm
- Organising help if complications occur
- Arranging social support
- Recovery expectations
- Returning to daily activities
- Returning to driving and other types of travel
- Returning to work
- Returning to sport/leisure activities
- Pain expectations
- Pain management
- Pain science education
- Rehabilitation after surgery
- Purpose of pre-habilitation
- Physical activity
- Weight management
- Weight loss
- Swelling
- Smoking/alcohol cessation
- Nutrition
- Goal setting
- Use of equipment/aids
- Anatomy
- Don’t know
- Other
  - Please specify ‘Other’: *[Required]*

**Section 3: Pre-habilitation while on the waiting list for knee replacement**

1. At your hospital, are patients provided with pre-rehabilitation (e.g. physiotherapy, hydrotherapy, pain management, Occupational Therapy, nutrition) while on the waiting list for knee replacement? *[Required]*

- Yes, provided to all patients
- Only provided to patients who meet certain criteria
  - Please provide details: *[Required]*
- No *[skip to Section 4]*
- Don’t know *[skip to Section 4]*

**S3.1: Pre-habilitation while on the waiting list for knee replacement**

1. At what time points would this pre-habilitation be provided?

- At the point of listing
- Between listing and consent and/or pre-operative assessment appointment
- Between consent and/or pre-operative assessment appointment and surgery
- Don’t know
- Other
  - Please specify ‘Other’: *[Required]*

1. Where is it provided? (Please tick all that apply)

- Hospital outpatients
- Community
- Home
- Don’t know
- Other
  - Please specify ‘Other’: *[Required]*

1. What is the format of the pre-habilitation? (Please tick all that apply)

- Group-based class
- Individual session
- Home visit
- Telephone/videocall
- Unsupervised home exercise programme
- Written information
- Don’t know
- Other
  - Please specify ‘Other’: *[Required]*

1. What is the average number of treatment sessions?

- <2
- 2-4
- 5-6
- >6
- Don’t know
- Other
- Please specify ‘Other’: *[Required]*

1. What treatment modalities are used? (Please tick all that apply)

- Advice
- Exercises
  - Please specify ‘Exercises’: *[Required]*
    - Strengthening exercises
    - Balance exercises
    - Flexibility exercises
    - Practicing post-operative exercises
    - Functional movement exercises (including gait re-education etc)
    - Training on steps
    - Functional technique exercises
    - Walking practice with walking aids
    - Core control exercises
    - Cardiovascular exercises
- Manual therapy (including soft tissue techniques)
- Electrotherapy
- Ice/Heat
- Acupuncture
- Hydrotherapy
- Pain management (including Cognitive Behavioural Therapy)
- Weight programme referral
- Cognitive Behavioural Therapy for formally diagnosed depression or anxiety
- Occupational Therapy
- Nutrition
- Don’t know
- Other
  - Please specify ‘Other’: *[Required]*

1. Are aids and/or specific equipment provided to patients for use prior to surgery?

- Yes
  - Please provide details of the aids/equipment provided *[Required]*
  - Who provides the aids/equipment? *[Required]*
    - Hospital
    - Outside provider
      - Please provide details: *[Required]*
- No
- Don’t know

1. At what time points are patients seen in the pre-operative assessment clinic prior to surgery?

- 1 weeks pre-operative
- 2 weeks pre-operative
- 3 weeks pre-operative
- Don’t know
- Other
  - Please specify ‘Other’: *[Required]*

**Section 4: Pre-habilitation while on the waiting list for hip replacement**

1. At your hospital, are patients provided with pre-habilitation (e.g. physiotherapy, hydrotherapy, pain management, Occupational Therapy, nutrition) while on the waiting list for hip replacement? *[Required]*
   - Yes
   - No *[skip to Section 5]*
   - Don’t know *[skip to Section 5]*

**S4.1: Pre-habilitation while on the waiting list for hip replacement**

1. Is the **pre-habilitation** provided to patients while on the waiting list for hip replacement **different** to that provided to patients waiting for knee replacement? *[Required]*

- Yes
- No *[skip to Section 5]*
- Don’t know *[skip to Section 5]*

**S4.2: Pre-habilitation while on the waiting list for hip replacement**

1. At what time points would this pre-habilitation be provided?

- At the point of listing
- Between listing and consent and/or pre-operative assessment appointment
- Between consent and/or pre-operative assessment appointment and surgery
- Don’t know
- Other
  - Please specify ‘Other’: *[Required]*

1. Where is it provided? (Please tick all that apply)

- Hospital outpatients
- Community
- Home
- Don’t know
- Other
  - Please specify ‘Other’: *[Required]*

1. What is the format of the pre-habilitation? (Please tick all that apply)

- Group-based class
- Individual session
- Home visit
- Telephone/videocall
- Unsupervised home exercise programme
- Written information
- Don’t know
- Other
  - Please specify ‘Other’: *[Required]*

1. What is the average number of treatment sessions?

- <2
- 2-4
- 5-6
- >6
- Don’t know
- Other
  - Please specify ‘Other’: *[Required]*

1. What treatment modalities are used? (Please tick all that apply)

- Advice
- Exercises
- Please specify ‘Exercises’: *[Required]*
  - Strengthening exercises
  - Balance exercises
  - Flexibility exercises
  - Practicing post-operative exercises
  - Functional movement exercises (including gait re-education)
  - Training on steps
  - Functional technique exercises
  - Walking practice with walking aids
  - Core control exercises
  - Cardiovascular exercises
- Manual therapy (including soft tissue techniques)
- Electrotherapy
- Ice/Heat
- Acupuncture
- Hydrotherapy
- Pain management (including Cognitive Behavioural Therapy)
- Weight programme referral
- Cognitive Behavioural Therapy for formally diagnosed depression or anxiety
- Occupational Therapy
- Nutrition
- Don’t know
- Other
  - Please specify ‘Other’: *[Required]*

1. Are aids and/or specific equipment provided to patients for use prior to surgery?

- Yes
  - Please provide details of the aids/equipment provided *[Required]*
  - Who provides the aids equipment? *[Required]*
    - Hospital
    - Outside provider
      - Please provide details: *[Required]*
- No
- Don’t know

1. At what time points are patients seen in the pre-operative assessment clinic prior to surgery?

- 1 weeks pre-operative
- 2 weeks pre-operative
- 3 weeks pre-operative
- Don’t know
- Other
  - Please specify ‘Other’: *[Required]*

**Section 5. Gaps in service provision**

1. Are there any services that are not currently provided within your hospital that you would like to see offered to patients waiting for hip and knee replacement? *[Required]*

- Yes
  - Please provide details: *[Required]*
- No
- Don’t know

1. Why do you think these services are not currently available? What do you think the barriers or facilitators are to providing this/these service(s)?

**Further Comments**

If you have any further comments, please write them in the box below:

**Thank You**

Thank you for helping us with our survey.

If you would like to contact us, please email us at *[EMAIL]*.
